# Supplementary material for: AlphaDesign: a de novo protein design framework based on AlphaFold
Source: Mol Syst Biol. 2025 Jun 17;21(9):1166–89. doi: 10.1038/s44320-025-00119-z (PMC12405559; doi:10.1038/s44320-025-00119-z)
Supplement: Supplementary file 1 — Appendix [file 44320_2025_119_MOESM1_ESM.pdf]

Appendix

Table of Contents

Table of Contents **1**
Appendix Figures **1**
Appendix Figure S1 – Computational cost of AlphaDesign. 1
Appendix Figure S2 – Computational analysis of structural flexibility and molecular contact dynamics for binder designs. 2
Appendix Figure S3 – normalised colony opacity measurements for designed RcaT-Sen2 binders. 3
Appendix Figure S4 – Circular dichroism spectroscopy measurements of RcaT-Sen2 inhibitor designs. 4
Appendix Figure S5 – SEC-MALS of RcaT-Sen2 inhibitor designs. 5
Appendix Figure S6 – Experimental validation of design cpx-50-nr2_run_5_0 by long-range NOEs. 6
Appendix Tables **7**
Appendix Table S1 – De novo RcaT inhibitor sequences. 7
Appendix Table S2 – Structure statistics for NMR ensemble of 100aa_3_1. 9
Supporting Methods **10**
AlphaDesign design loop pseudocode 10
Sequence design pseudocode 12

Appendix Figures

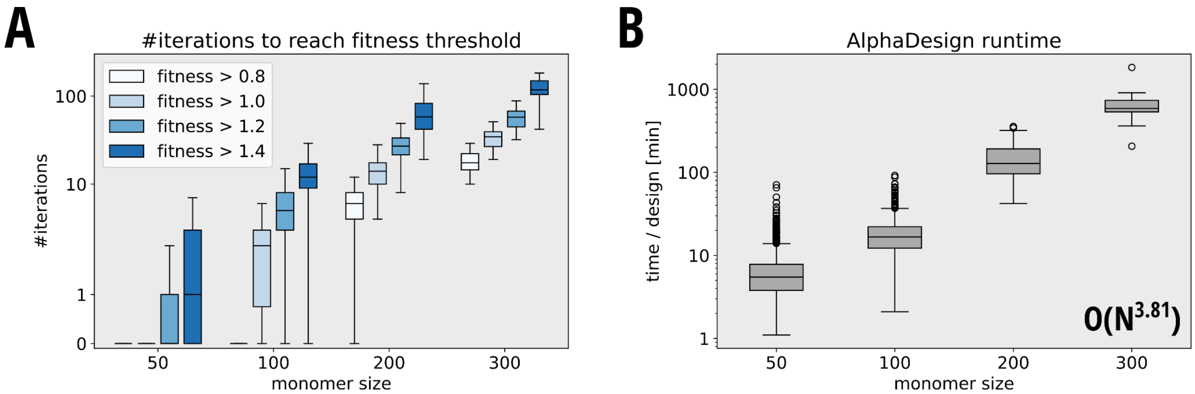

Appendix Figure S1 – Computational cost of AlphaDesign.

A Boxplot showing the number of AlphaDesign iterations needed for AlphaDesign to reach a given fitness threshold on 50 to 300 amino acid monomers. Boxes are coloured by fitness threshold. The centre line of each box corresponds to the median, box edges to the upper and lower quartiles, and whiskers to the lowest and highest data points excluding outliers (within 1.5 times the interquartile range above or below the box edges). Sample sizes for

each monomer size are as follows: 50 AA: N = 1037; 100 AA: N = 540; 200 AA: N = 75; 300 AA: N = 29.

B Boxplot showing the distribution of time required per design in minutes for 50 to 300 amino acid monomers. Median computation time scales approximately as $O(N^{3.81})$ with the total number of amino acids N. Sample sizes, boxes, centre line and whiskers as in (A), outliers above and below the range of the whiskers are shown as circles.

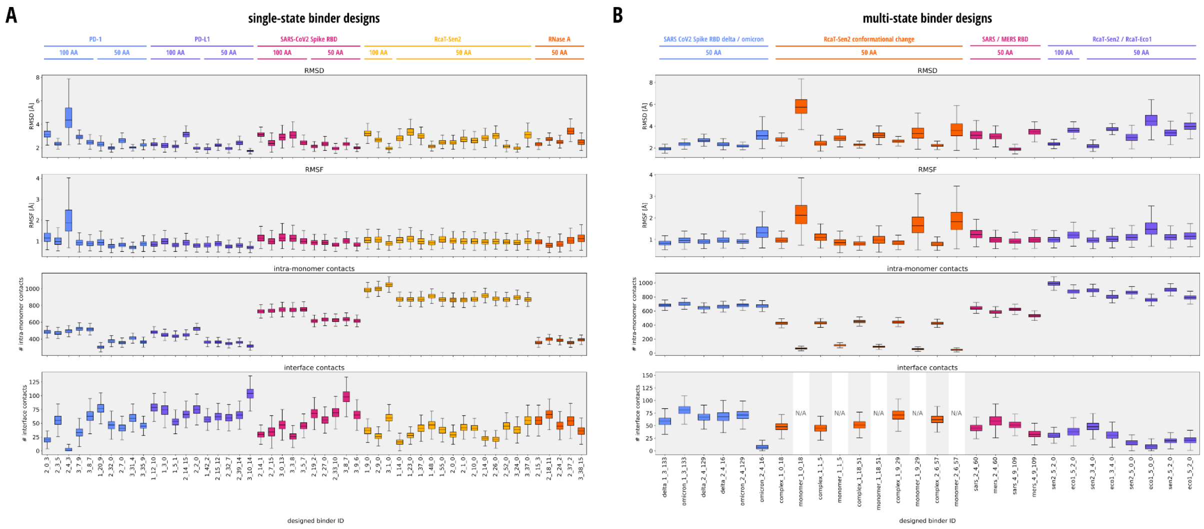

Appendix Figure S2 – Computational analysis of structural flexibility and molecular contact dynamics for binder designs.

A, B Boxplots of the distribution of computed structural flexibility and molecular contact properties from molecular dynamics simulation ensembles for (A) single-state and (B) multi-state binder designs. RMSD relative to the predicted conformation, RMSF across the entire ensemble, number of intramolecular and intermolecular contacts are shown for N = 20,000 snapshots. Non-applicable statistics such as intermolecular contacts for monomeric systems are marked as N/A. The majority of simulated binder designs exhibit low RMSD and RMSF, with a high number of inter and intramolecular contacts. This indicates that the majority of designs are stable. In (B) many binders designed to change conformation exhibit high RMSD and RMSF, which drops significantly in the complex state. This is indicative of a flexible structure in the monomeric state that transitions to a more rigid (low RMSD, RMSF) structure in the complex state. The centre line of each box in the box plot corresponds to the median, box edges to the upper and lower quartiles, and whiskers to the lowest and highest data points excluding outliers (within 1.5 times the interquartile range).

Appendix Figure S3 – normalised colony opacity measurements for designed RcaT-Sen2 binders.

Bar height is given by the mean over n=6 replicates (n=16 replicates for the GFP control), error bars by their standard deviation.

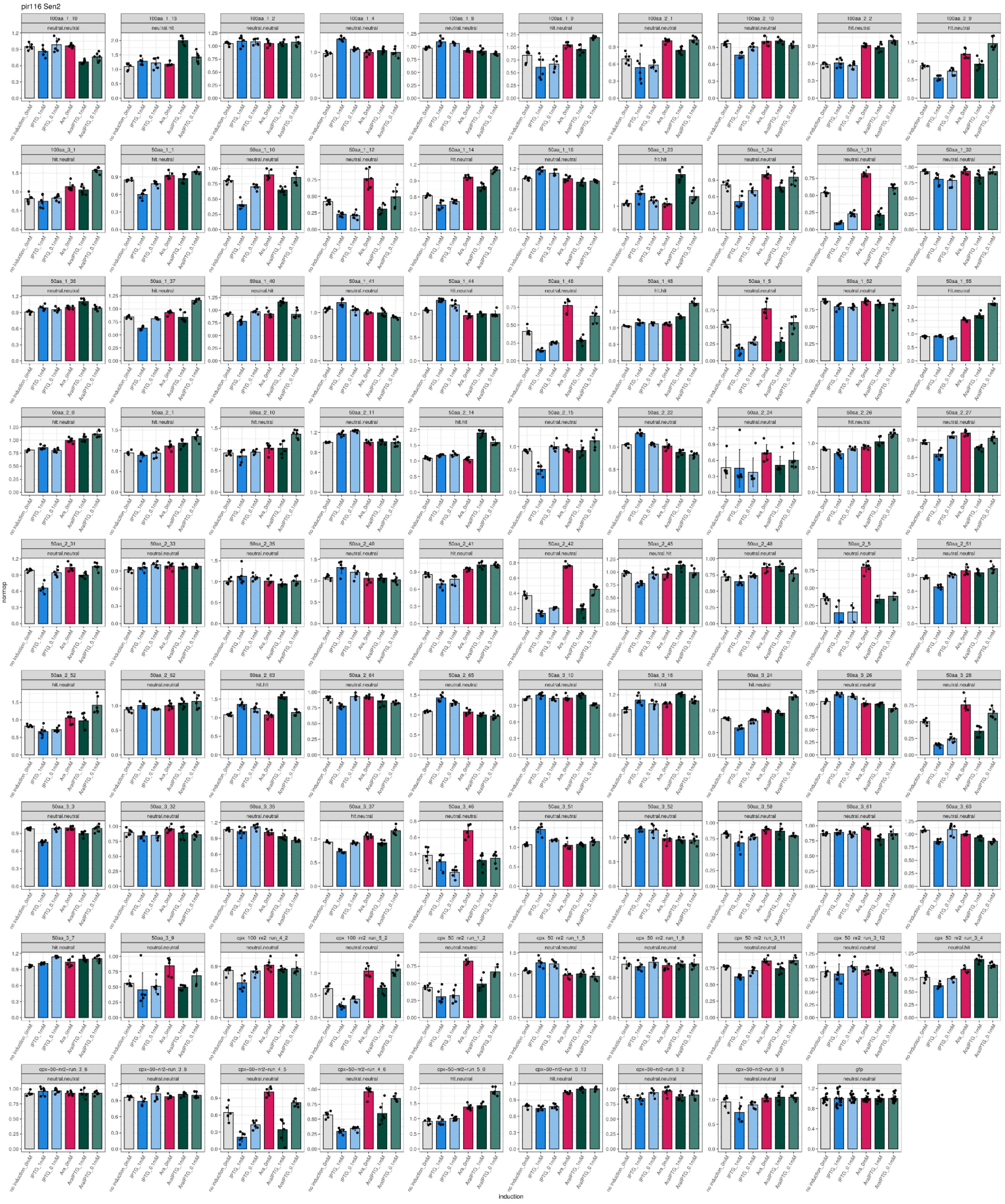

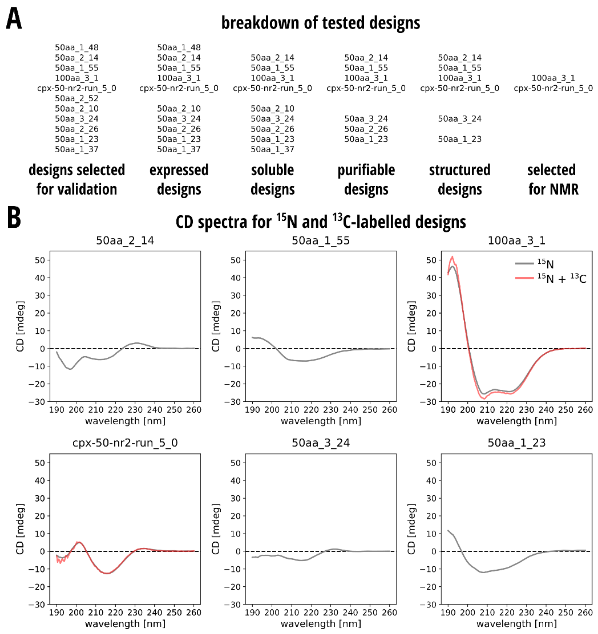

C

CD secondary structure content

Design		α-helix	β-sheet	Turn	Coils	Other
50aa_2_14	Jasco	8.6%	46.9%	10.6%		34.0%
	BeStSel	0.0%	47.6%	13.4%		39.0%
	CDSSTR	0.0%	44.0%	20.0%		35.0%
	PDBM2CD prediction	5.8%	48.1%	15.4%		30.8%
	KCD prediction	0.0%	46.2%		23.1%	30.8%
50aa_1_55	Jasco	24.2%	29.4%	10.9%		35.5%
	BeStSel	12.6%	28.3%	13.6%		45.6%
	CDSSTR	16.0%	30.0%	21.0%		34.0%
	PDBM2CD prediction	30.8%	23.1%	5.8%		40.4%
	KCD prediction	30.8%	23.1%		30.8%	15.4%
100aa_3_1	Jasco	34.5%	12.3%	14.6%		38.6%
	BeStSel	35.6%	16.9%	13.2%		34.4%
	CDSSTR	48.0%	11.0%	13.0%		29.0%
	PDBM2CD prediction	70.6%	0.0%	7.8%		21.6%
	KCD prediction	55.9%	0.0%		24.5%	19.6%
cpx-50-nr2 run_5_0	Jasco	7.4%	62.0%	7.9%		22.8%
	BeStSel	1.0%	52.7%	11.4%		34.9%
	CDSSTR	0.0%	44.0%	29.0%		26.0%
	PDBM2CD prediction	0.0%	73.1%	7.7%		19.2%
	KCD prediction	0.0%	74.9%		7.7%	15.4%
50aa_3_24	Jasco	21.0%	32.2%	12.3%		34.6%
	BeStSel	0.0%	43.8%	12.8%		43.5%
	CDSSTR	2.0%	41.0%	20.0%		35.0%
	PDBM2CD prediction	0.0%	44.2%	13.5%		42.3%
	KCD prediction	0.0%	44.2%		30.8%	25.0%
50aa_1_23	Jasco	23.5%	31.0%	11.2%		34.4%
	BeStSel	14.5%	32.4%	14.1%		39.0%
	CDSSTR	8.0%	31.0%	20.0%		40.0%
	PDBM2CD prediction	25.0%	34.6%	3.9%		36.5%
	KCD prediction	13.5%	34.6%		36.5%	15.4%

Appendix Figure S4 – Circular dichroism spectroscopy measurements of RcaT-Sen2 inhibitor designs.

A Overview which designs were selected for validation and passed subsequent stages of validation. Of 11 tested designs, 10 were expressed in E. coli, of which 9 were soluble as His_6-Sumo3 fusion proteins. Of these 9 designs, 7 remained soluble after removal of the His_6-Sumo3 fusion tag. Of these 6 appeared structured in initial CD screening. CD spectra for these are shown in (B). Of these 6 designs, two were selected for structure validation using NMR.

B Circular dichroism spectra for 6 selected RcaT-Sen2 inhibitors. CD spectra in grey were recorded for -labelled proteins, and spectra in red were recorded for double-labelled proteins which were selected for NMR structure determination.

C Table of predicted and calculated secondary structure contents of each design using CD spectroscopy. For each design, the secondary structure contents derived from CD data using the Multivariate SSE analysis of the Jasco Spectra Manager software (Jasco), BeStSel (Micsonai et al, 2018) and CDSSTR (Johnson, 1999) are listed together with predictions for the designed structure using PDBM2CD (Mavridis & Janes, 2016) and (Jacinto-Méndez et al, 2024). Secondary structure content derived from CD data is in good agreement for all designs.

A

SEC-MALS of purified designs

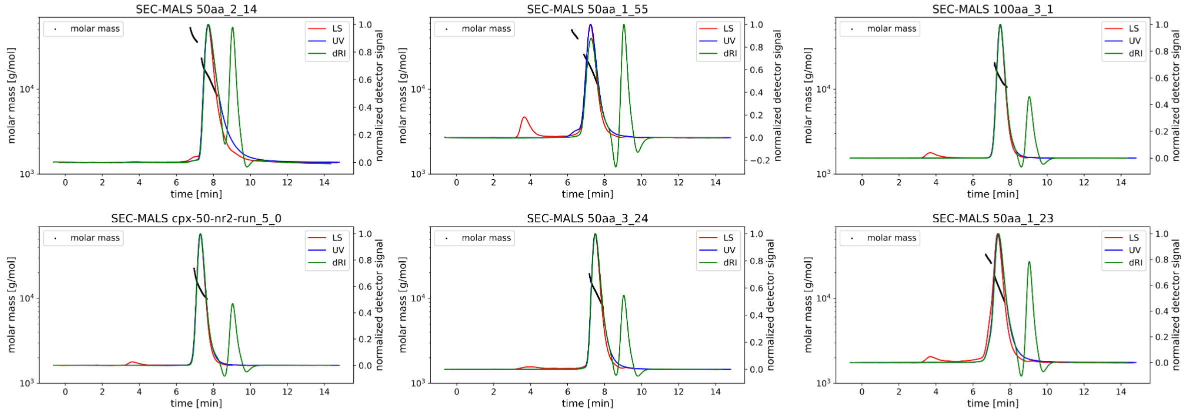

B

SEC-MALS summary table

	Peak 1			Peak 2		
	Mw (kDa)	Polydispersity (Mw/Mn)	Mass fraction (%)	Mw (kDa)	Polydispersity (Mw/Mn)	Mass fraction (%)
50aa_2_14	13.3 (±4.9%)	1.054 (±6.689%)	99.2	40.8 (±1.8%)	1.010 (±2.475%)	0.8
50aa_1_55	17.5 (±4.2%)	1.047 (±5.690%)	99.6	43.2 (±3.1%)	1.004 (±4.425%)	0.6
100aa_3_1	13.4 (±3.4%)	1.024 (±4.436%)	100.1			
cpx-50-nr2-run_5_0	12.9 (±4.6%)	1.031 (±5.867%)	100			
50aa_3_24	11.8 (±4.4%)	1.041 (±5.914%)	99.9			
50aa_1_23	13.4 (±4.8%)	1.041 (±6.307%)	91.5	28.4 (±7.2%)	1.004 (±10.200%)	8.2

Appendix Figure S5 – SEC-MALS of RcaT-Sen2 inhibitor designs.

A Normalized detector signal for light scattering (LS), differential refractive index (dRI) and UV absorbance (UV) over time (right y-axis), together with molar mass (left y-axis) for all peaks.

B Determined molecular weight for SEC each peak, polydispersity and mass fraction for each SEC peak in the SEC-MALS chromatogram, for each tested design.

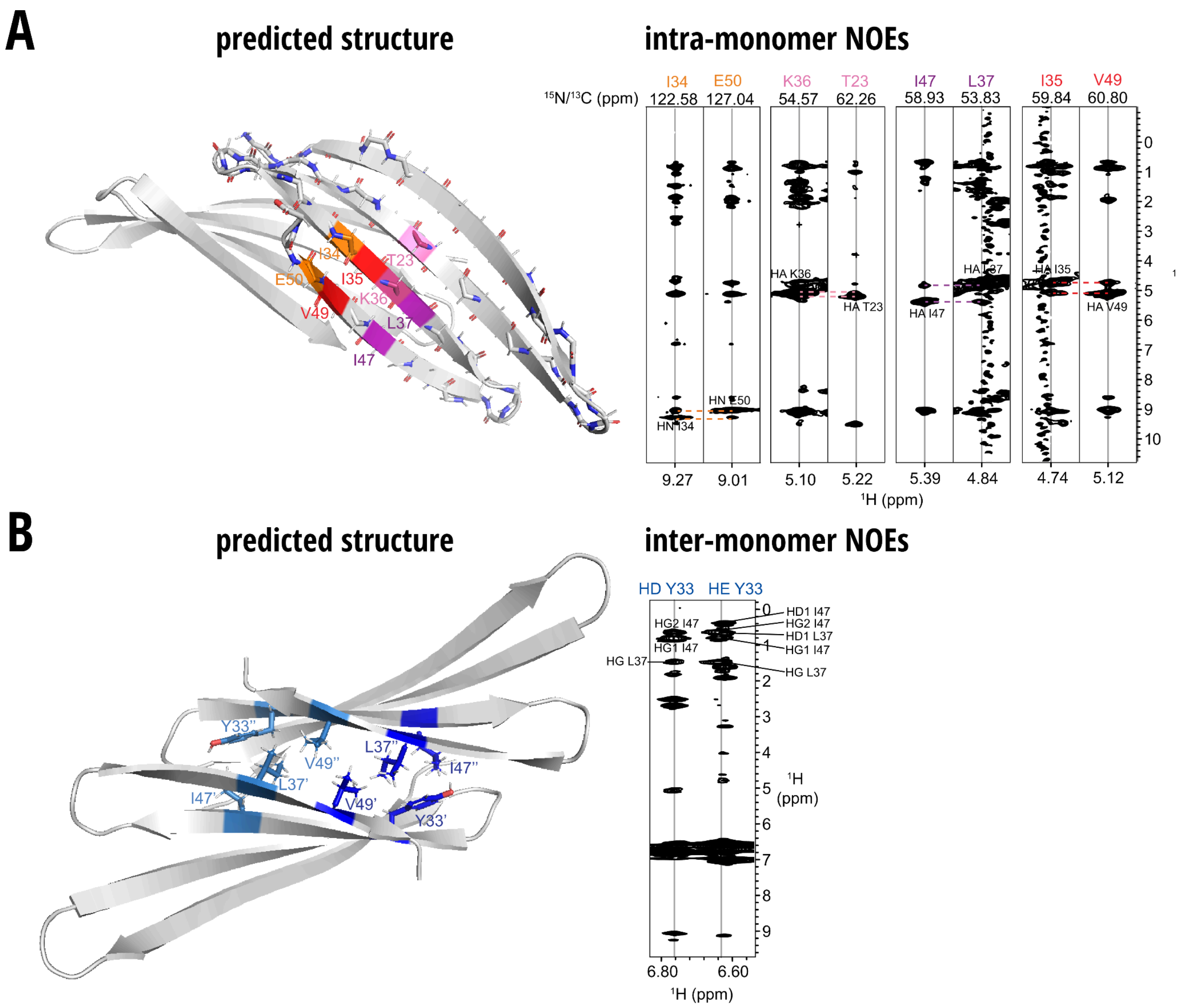

Appendix Figure S6 – Experimental validation of design cpx-50-nr2_run_5_0 by long-range NOEs.

A The β-strand arrangement is validated by H_N-H_N and H_α-H_α-NOEs of adjacently located residues between two β-strands. Residue labels of NOE strips are colored according to their color in the structure (left) for better orientation. The NOE-pairs are highlighted in the NOE strips and indicated by a dashed line of the same color as the corresponding residue. The ^{15}N-terminal residues exchange on a time scale, which leads to severe line broadening and are therefore not visible. Nevertheless, from secondary chemical shifts (Figure 7 G) and these NOEs, the β-strand arrangement is confirmed.

B The long-range NOEs between Y33 and residues involved in the dimerization interface (L37, I47 and V49) are shown next to the structure. Especially NOEs between Y33' and I47''/L37'' are only possible upon dimerization. Accordingly, long-range NOEs confirm this dimerization motif.

Appendix Tables

Appendix Table S1 – De novo RcaT inhibitor sequences.

A list of lengths, design IDs, inhibitor activities against RcaT-Sen2 and RcaT-Eco9 as well as sequences of successful RcaT inhibitor designs.

#AA	id	Sen2 activity	Eco9 activity	leaky	sequence
50	1_48	1.57	0.98	✓	MEIVIIREKLDLYGLDIDYVEIIYSKSKENRYFGI KLIKDDKIIKTIGEM
50	2_14	1.51	1.16	×	SMPLSEFDRLAGFLKRNDKFYLVYEKNGKYY AIGPDNEIILVDKNGNIIP
50	1_55	1.40	1.38	✓	GDMNIPVFVIKLPKPPPEEVLPRLIGTYEKML KELYKKPYKLEVIIEEPE
50	2_52	1.34	1.31	×	MLKGVEVTLEIKIDNEDKLIVQKITVKFIYTEEG FKNEIKVEIIEDPSEE
50	2_10	1.33	1.09	×	MEKPKVELYEKETEEGKILVAIFQYPDKEEPE FIVATDEKEIEELKKKAE
50	3_24	1.31	1.08	×	KRDNFYEEFILNNKKIIKIYLDEKIPGEPPRPLK VYYLDAETGELLTDLP
50	2_26	1.28	0.73	×	KYPILEKVTSEGGYTTILFKEPVTEEEIKEIKSD TKIFVATLKEPPKLYE
50	1_23	1.27	1.13	×	MSEELKEYLKKNLPKEYGPYTGKFGDLELFIV PLEEGWDLDKLYILEIKP
50	1_37	1.25	1.20	×	MEEEIKELTDKLIKMFEENKLYSLDLTFEDALE KMPEFNELLKELKEKKP
50	2_1	1.21	1.16	×	MEEEEKKKKKEEELEELKKELKSLDISKLTPE EFKKFYFEVLKKYLELKK
50	1_14	1.14	1.30	×	MKIKKIIVNAPKLPDGGYEGIAVIILENGKSIIAY AKGKGYISPNIPPGE
50	2_0	1.12	1.08	×	EIIEVELLDVEGKKKIVLKLNEEEYKVYKVVRE MLKAAEKGEKADVEKLL
50	3_37	1.10	1.30	×	VEEGLKEGKIGEARKKRIEEDIKILEEIIKSGNL EKFFEYLKEKLEEEKK

50	1_1	1.07	1.34	×	MEKEAKEYYKELVEWYKKLEEENKGGKGLT EEEKEKKIEERLKELEEKRK
50	1_16	0.95	1.35	×	MSGPVLKIVIKAENEEEAEELKEKYKKEIEGRI KRGRWPELKLEFEIELL
50	2_27	0.91	1.73	×	EELKKIMEEAKEKIKNNEELSEELIKKYLELMN KIRELPEEEQEKILKEI
50	3_10	0.88	1.33	×	LTPKEKLEKEIEEFREEYEKVWDKLGPMTKE MIEKAIEYAKEEFKKKYPE
50	3_28	0.83	1.32	×	SEEDKKKIKELYEKYYNPNNTEEEKKEILKEW EEYIKEKTKKEGKEEYLK
50	1_46	0.81	1.50	×	LFEELKKIIPELLKGAKSEGEALRIIQKATIEGL KKLYELEKKKKEEEEK
50	2_5	0.46	1.77	×	KEEFKKKLNEFLKYFRENFPKLFPDATEEDF GKFFLELAKKIVETPEEEI
50	cpx-50-nr2-run_5_0	1.37	3.0	✓	MIINKGIEEVQEPDKTVLVETLIIEGEDGEYIIKL IKYPDGSSRIEVEKK
100	3_1	1.39	1.27	✓	DKEWISKLPKSPEPWTPEQEEAFLKRFAEKV DPEETLKKLEEWIKENIKK YPEYKDELEVAYNSAKLFLESPLVEGPGKVR AIGRVLWTIKRLNIDSPFV
100	2_9	1.24	1.30	×	GKIEIKEIAENVLEIKINENIKPNEKKIIYIFRDDI GLYIILESGNHNFT IIFVDDDKVIVKLPSPKNNKNQKYHVVIGKYPV KITEEGDYLIATNEIKK
100	1_9	1.14	1.09	✓	EPSLVEKVKEYLKKNPPKTLEEKKILESLMIAA ENQEKIDEILEKYKPKG ELPEEVKKEILENKQIINLSVINVESLLEKLDKS IFPEWLLEEVKKLKEY

Appendix Table S2 – Structure statistics for NMR ensemble of 100aa_3_1.

**Conformational restraints**	
NOE distance restraints	
total	1389
short range $\|i - j\| \leq 1$	802
medium range $\|i - j\| < 5$	337
long range $\|i - j\| \geq 5$	250
ϕ/ψ dihedral restraints from TALOS (Shen *et al*, 2009)	60/62
Hydrogen bond restraints (upper / lower)	92/92
**Structure statistics**	
CYANA (Güntert, 2009) target function [$Å^2$]	1.6
# NOE violations > 0.2 Å	1
Maximum NOE violation [Å]	0.28
Dihedral angle violations > 4°	0
Ramachandran plot statistics<br>generously allowed / disallowed regions (%)	81.6 / 14.8 / 2.8 / 0.7
Coordinate precision (RMSD to mean coordinates)	
Backbone (A24 - S99) [Å]	0.5
Heavy atoms [Å]	1.01

Supporting Methods

AlphaDesign design loop pseudocode

```
# function which generates a single protein candidate
def generate_candidate(
	blueprint, # description of sequence length and complex stoichiometry
	fitness, # fitness function assigning a real number to a given sequence
	population_size=10, # number of sequences in the population
	suboptimality=0.1, # maximum suboptimality percentage for selection
	multiplicity=2, # multiple of population size used during mutation
	threshold=1.0 # fitness threshold for a successful design
):
	# initialise population of sequences uniformly
	# at random from a blueprint describing sequence length
	# and oligomer stoichiometry.
	population = [
		random_sequence(blueprint)
		for i in range(10)
	]
	# while the maximum fitness is below the threshold:
	while maximum(fitness(population)) < threshold:
		# select the sequences in the population where
		# fitness(sequence) >= suboptimality * maximum(fitness(population))
		parents = select(population, fitness, suboptimality)
		# construct a new population of size multiplicity * population_size
		new_population = []
		while len(new_population) < multiplicity * population_size:
			# at each iteration: recombine parent sequences
			parents = recombine(parents)
			# update the population with a greedy
			# rollout on all parent sequences
			new_population += rollout(parents, fitness)
		# trim population down to population_size
		population = top_k(new_population, k=population_size)
		# return the sequence with the highest fitness
		return sequence_with_max_fitness(population, fitness)

# selects the parents according to a fitness function
# and suboptimality percentage
def select(population, fitness, suboptimality):
	return [
		sequence
		for sequence in population
		if fitness(sequence) >= suboptimality * maximum(fitness(population))
	]

# greedy rollout for sequence improvement
def rollout(parents, fitness):
	outputs = []
```

```
# while we have parents with high fitness
while parents:
	# mutate these parents
	mutants = mutate(parents)
	# compute fitness for parents and mutants
	start_fitness = fitness(parents)
	mutant_fitness = fitness(mutants)
	# new parents in the rollout are mutants
	# with fitness higher than their parent
	parents = mutants[mutant_fitness > start_fitness]
	# append all mutants to the output
	outputs += mutants
return outputs
```

Sequence design pseudocode

```
# samples an amino acid sequence starting from a partially
# or fully masked sequence, a structure and a tie_index
# which ties together positions in the sequence
def sample_sequence(masked_sequence, structure, tie_index, temperature=0.1):
      while any_masked_positions(masked_sequence):
            masked_sequence = update_step(
                  masked_sequence, structure,
                  tie_index, temperature=0.1)
      return masked_sequence

# updates a single position in a sequence with a newly sampled
# amino acid.
def update_step(
      masked_sequence,
      structure,
      tie_index,
      temperature=0.1
):
      # get predicted logits from ADM, given a masked sequence and structure
      logits = ADM(masked_sequence, structure)
      # for homooligomers & repeat proteins, take the mean over all
      # tied positions given by tie_index.
      logits = mean_over_index(logits, tie_index)
      # scale logits by temperature
      logits = temperature * logits
      # transform logits into probabilities using softmax
      probabilities = softmax(logits)
      # sample a random masked position from the masked_sequence
      position = random_masked_position(masked_sequence)
      # sample a new amino acid from the categorical distribution
      # defined by probabilities
      new_amino_acid = random_categorical(probabilities[position])
      # set the masked sequence to new_amino_acid at position +
      # all tied positions and return
      masked_sequence[tie_index[position]] = new_amino_acid
      return masked_sequence
```
