# Supplementary figures and images for: AlphaDesign: a de novo protein design framework based on AlphaFold
Source: Mol Syst Biol. 2025 Jun 17;21(9):1166–89. doi: 10.1038/s44320-025-00119-z (PMC12405559; doi:10.1038/s44320-025-00119-z)

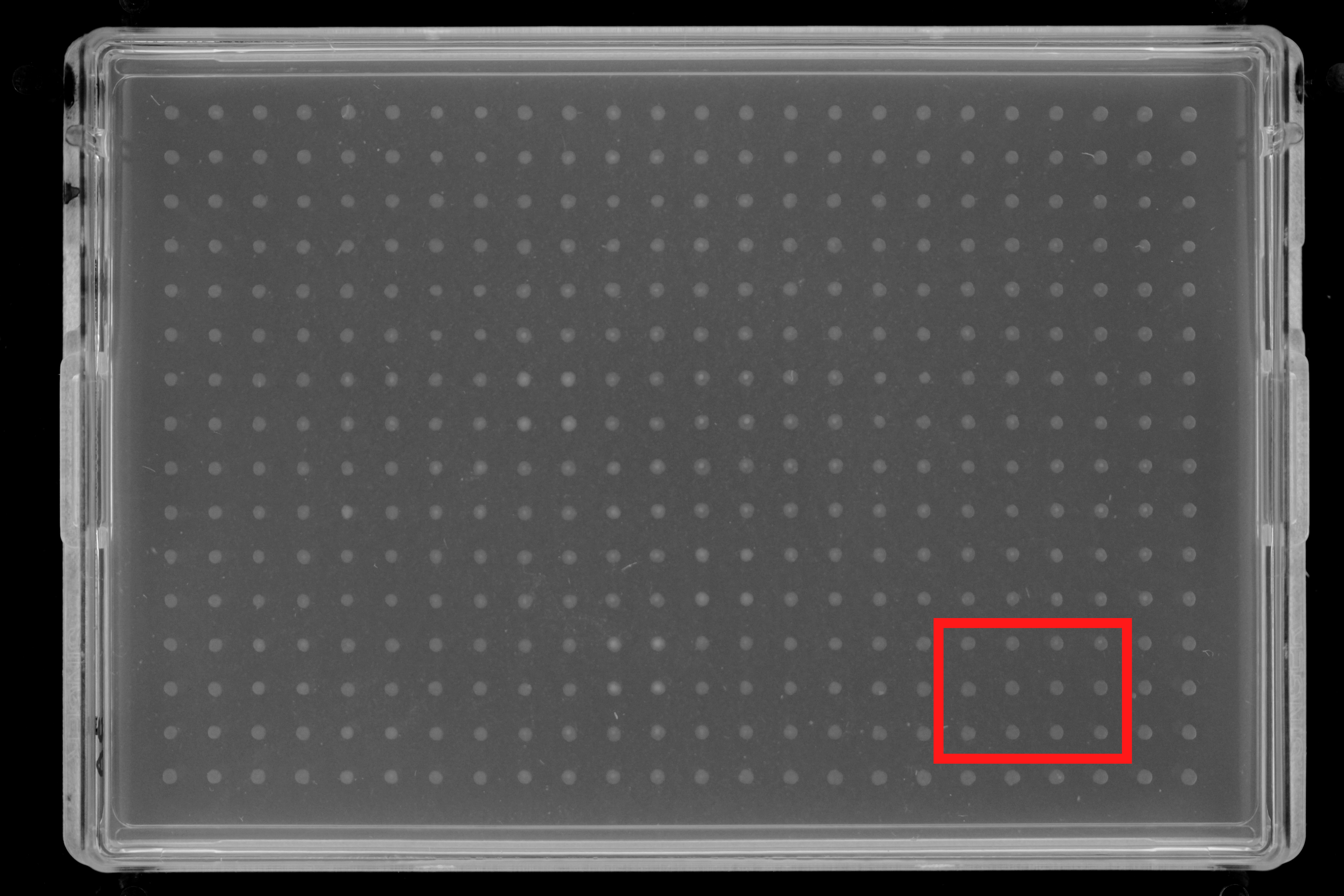

Supplement: Supplementary file 9 — Source data Fig. 7 [file 44320_2025_119_MOESM9_ESM.zip › Figure_7/7A/RcaT_Sen2_TIC_assay_RcaT_induction_plate_image_negative_control_crop.png]

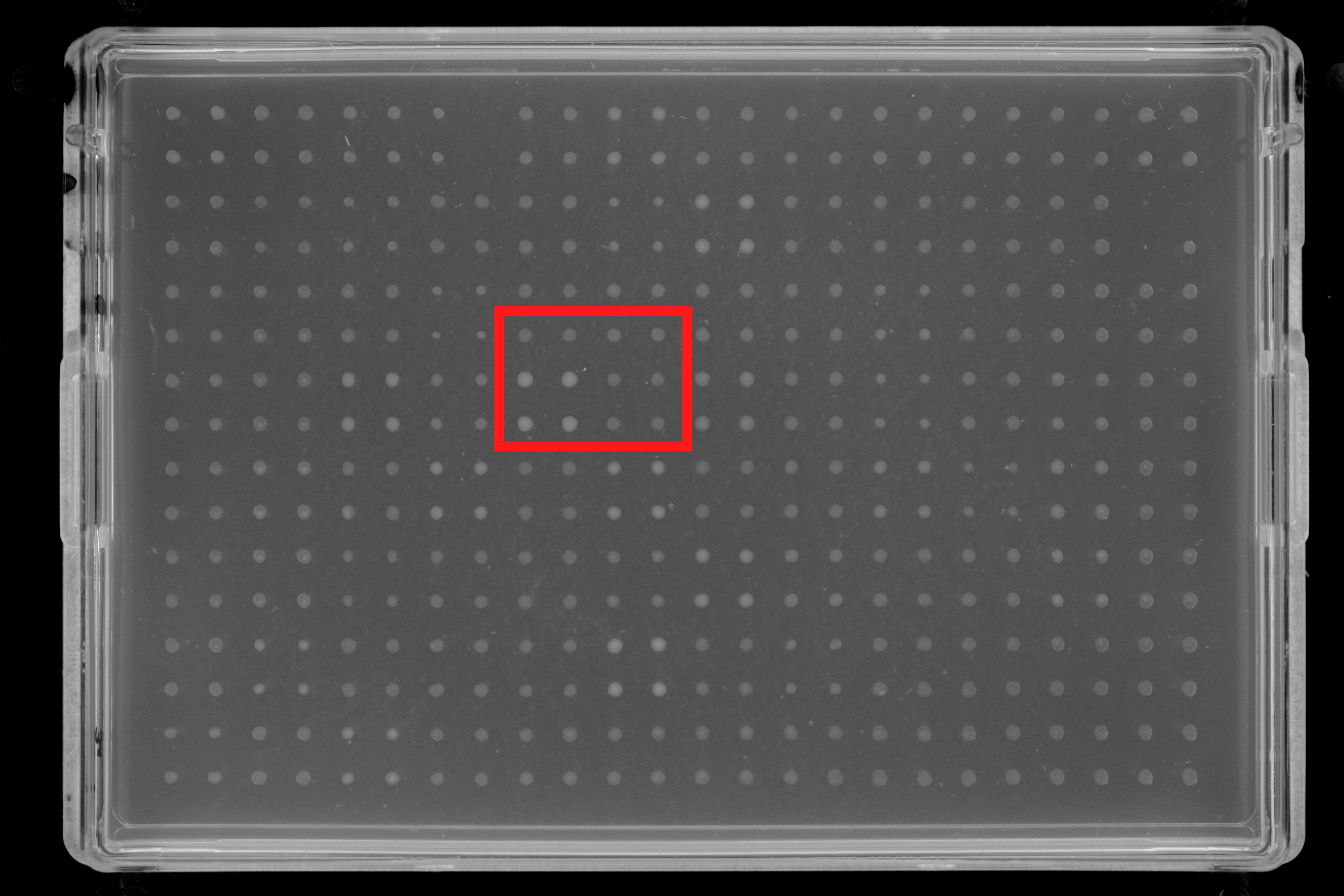

Supplement: Supplementary file 9 — Source data Fig. 7 [file 44320_2025_119_MOESM9_ESM.zip › Figure_7/7A/RcaT_Sen2_TIC_assay_double_induction_plate_image_designs_crop.png]

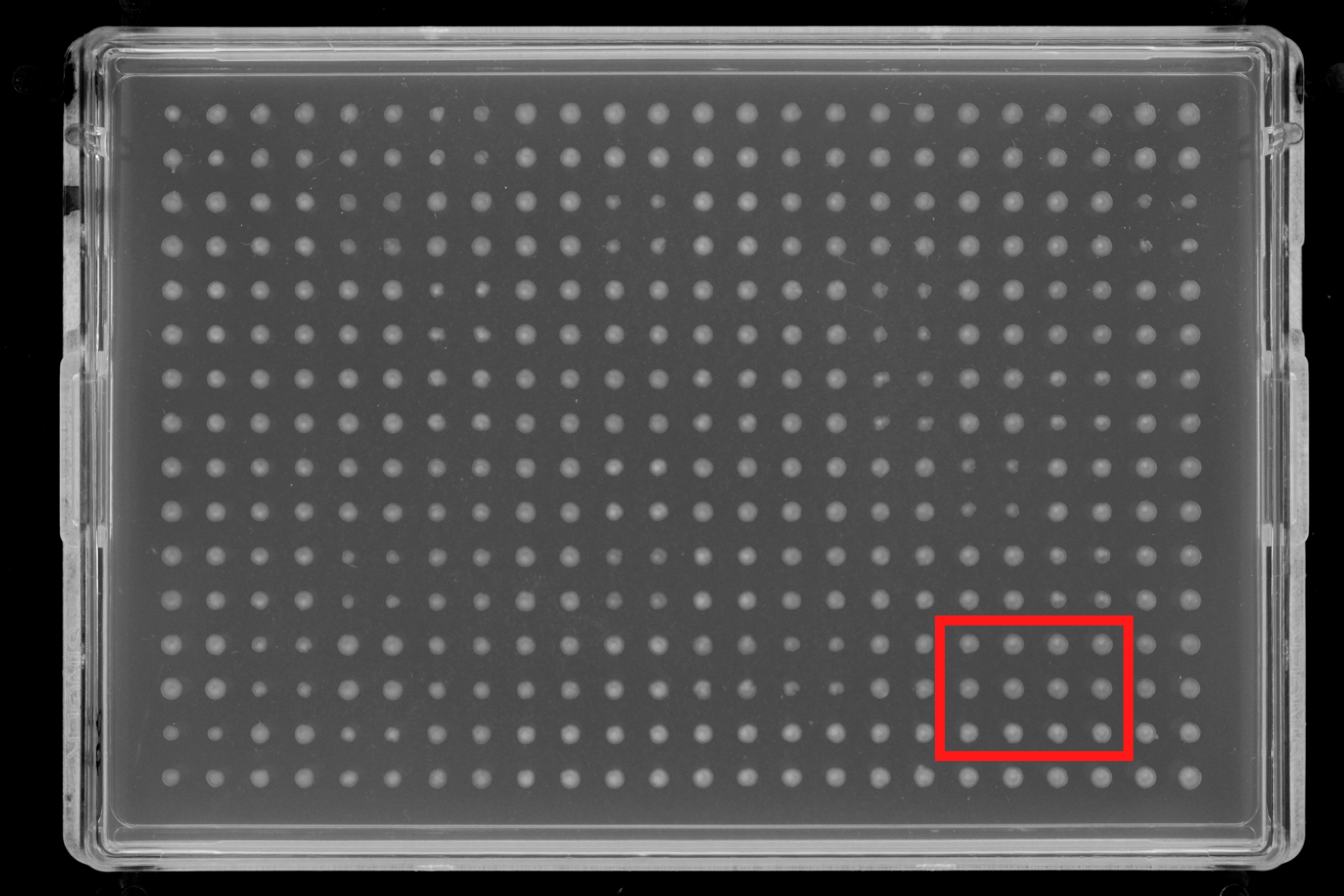

Supplement: Supplementary file 9 — Source data Fig. 7 [file 44320_2025_119_MOESM9_ESM.zip › Figure_7/7A/RcaT_Sen2_TIC_assay_no_induction_plate_image_positive_control_crop.png]
