# Supplementary material for: AlphaDesign: a de novo protein design framework based on AlphaFold
Source: Mol Syst Biol. 2025 Jun 17;21(9):1166–89. doi: 10.1038/s44320-025-00119-z (PMC12405559; doi:10.1038/s44320-025-00119-z)
Supplement: Supplementary file 10 — Expanded View Figures [file 44320_2025_119_MOESM10_ESM.pdf]

# Expanded View Figures

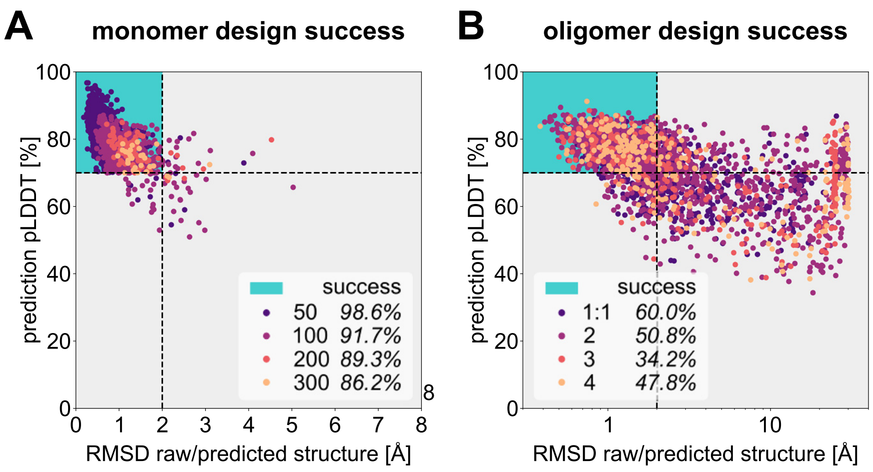


**Figure EV1. ESMfold de novo design success.**

(**A**, **B**) Scatter plots of redesigned sequence scRMSD/pLDDT using ESMfold for monomers (**A**) and oligomers (**B**). Designs are coloured by number of amino acids and percentages of successful designs are reported. The turquoise region and dotted lines indicate the region of success in terms of scRMSD/pLDDT (scRMSD < 2.0 Å, pLDDT > 70.0).

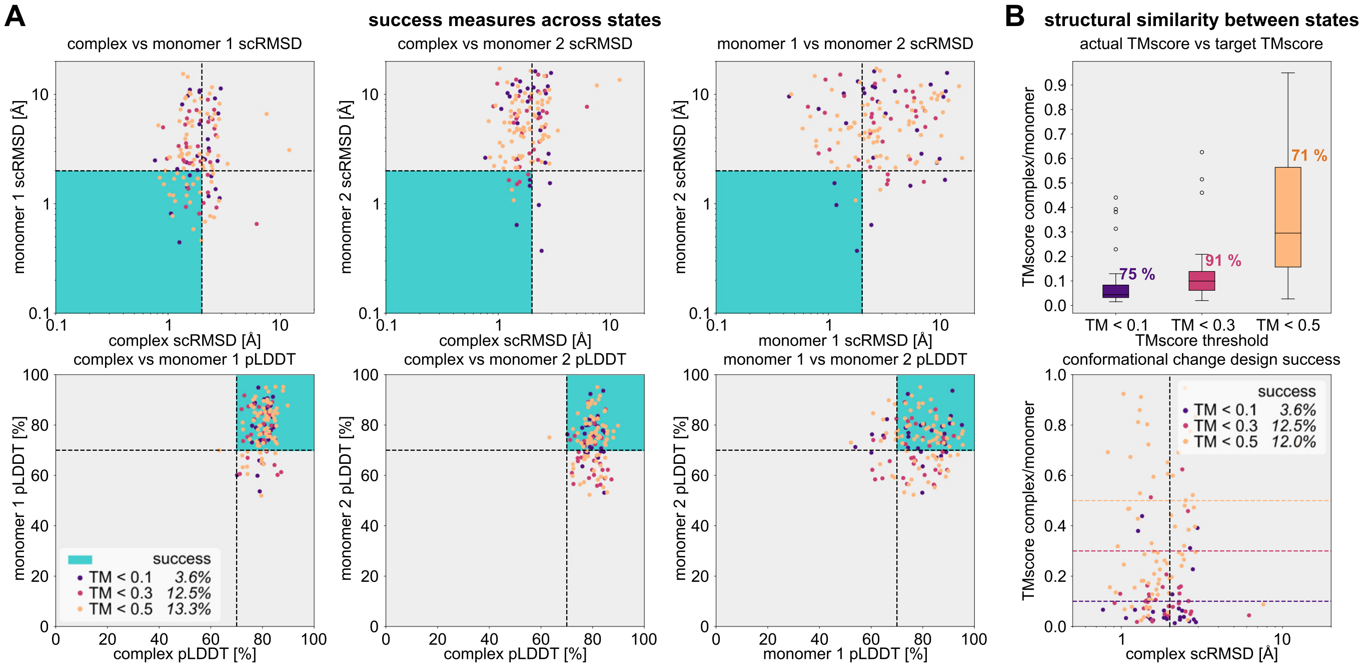


**Figure EV2. Conformational change design success.**

(**A**) Scatter plots of scRMSD (top) and pLDDT (bottom) using AlphaFold, comparing the complex state and both monomers of a conformational change design. Designs are coloured by their TM-score threshold (0.1, $N = 28$; 0.3, $N = 32$; or 0.5, $N = 75$) and the region of successful designs in terms of scRMSD or pLDDT is marked in blue. For each TM-score threshold, the percentage of successful designs with complex scRMSD < 2.0 Å, pLDDT > 70 and monomer scRMSD < 3.0 Å are reported. (**B**) Distribution of TM-scores between the complex and monomeric state predicted using AlphaFold for each TM-score threshold. (top) as a box-plot reporting the percentage of designs with TM-score below threshold. (bottom) as a scatter plot comparing scRMSD and TM-score. Designs are coloured by TM-score threshold and correspondingly coloured dotted lines mark the position of each threshold in the scatter plot (0.1, $N = 28$; 0.3, $N = 32$; 0.5, $N = 75$). The centre line of each box in the box plot corresponds to the median, box edges to the upper and lower quartiles, and whiskers to the lowest and highest data points excluding outliers (within 1.5 times the interquartile range).

 © The Author(s)

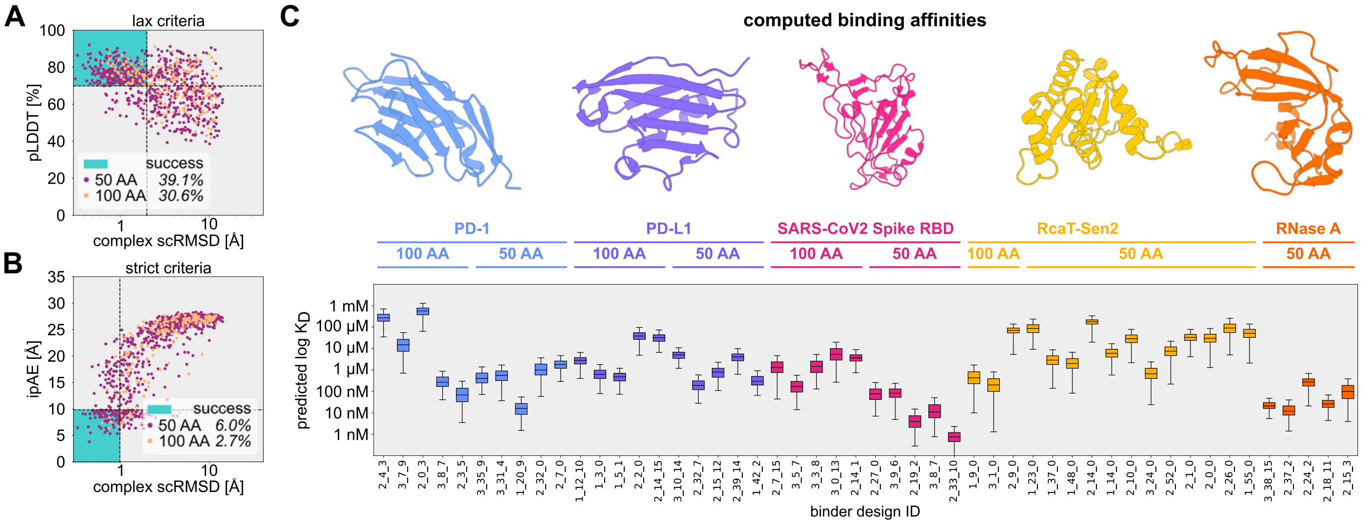


**Figure EV3. Binder design success rate.**

(**A**, **B**) Scatter plots of redesigned sequence scRMSD, pLDDT and interface pAE for binder designs to all target proteins considered in this work. Designs are coloured by number of amino acids (50 AA: $N = 715$, 100 AA: $N = 203$) and percentages of successful designs are reported. A shows lax success criteria, B shows strict success criteria. (**C**) Box plot of predicted dissociation constants for a set of selected binder designs over $N = 20{,}000$ molecular dynamics snapshots. Designs are grouped by target protein and the structures of each target are displayed above. The centre line of each box corresponds to the median, box edges to the upper and lower quartiles, and whiskers to the lowest and highest data points excluding outliers (within 1.5 times the interquartile range).

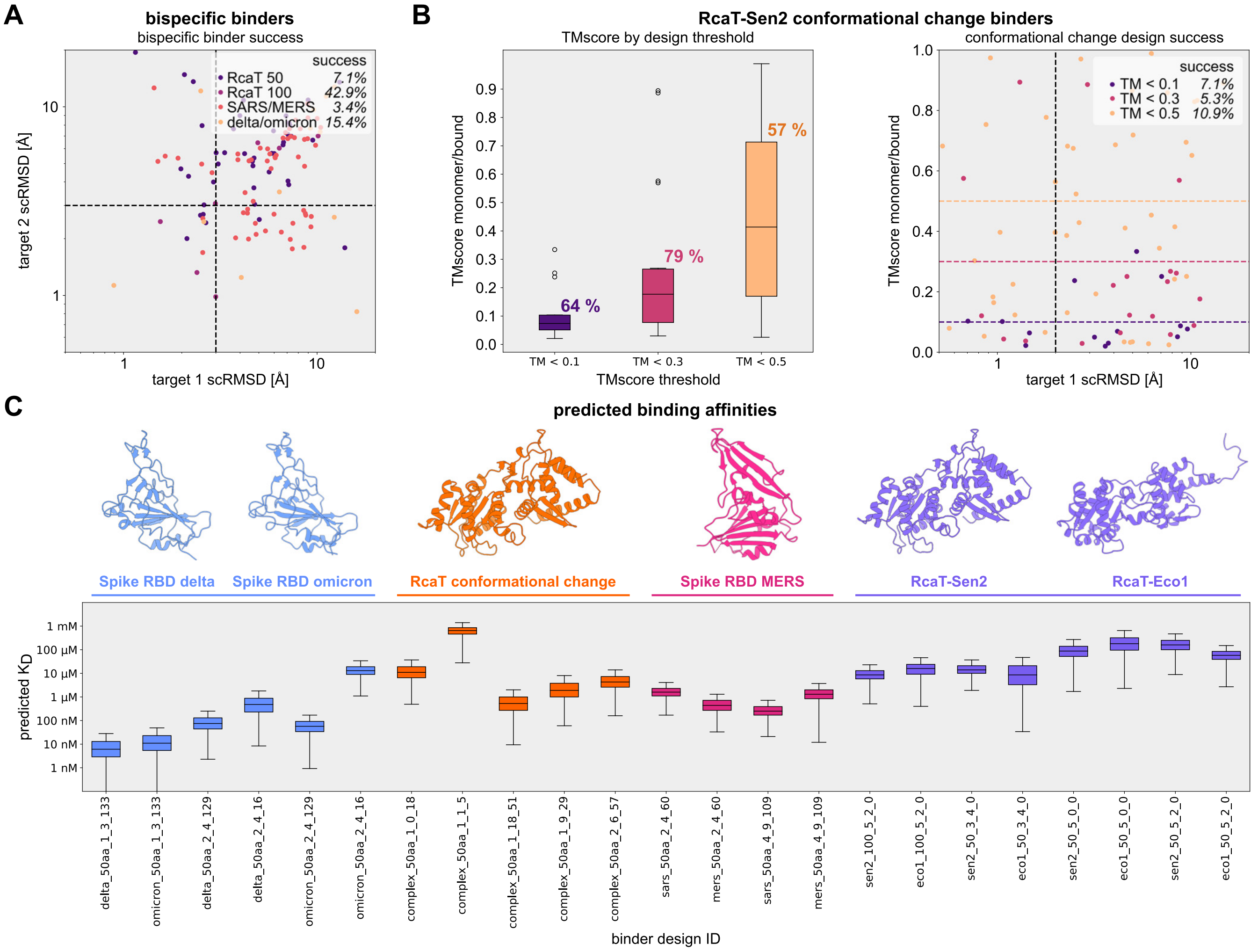


**Figure EV4. Multistate binder design.**

(**A**) Scatter plot of complex scRMSD for two targets of a bispecific binder design. Designs are coloured by target and number of amino acids (RcaT-Sen2/Eco1 50 AA: $N = 42$, 100 AA: $N = 7$; SARS/MERS Spike RBD 50 AA: $N = 58$; delta/omicron Spike RBD 50 AA: $N = 13$). Dotted lines represent a relaxed threshold for bispecific binder design success of 3.0 Å. Percentage of successful designs with pLDDT > 70 and scRMSD < 3.0 Å are reported for each pair of targets and designed binder size. (**B**) Statistics of proteins designed to change conformation upon RcaT-Sen2 binding. (left) Scatter plot of complex scRMSD of the bound state (x-axis) compared to the binder TM-score between the bound and unbound state. Designs are coloured by the maximum TM-score threshold used in their fitness function (TM < 0.1: $N = 14$, TM < 0.3: $N = 19$, TM < 0.5: $N = 46$). Coloured dotted lines represent these respective TM-score thresholds. The black dotted line represents the threshold for design success of scRMSD < 2.0 Å. Percentage of successful designs per TM-score threshold is reported. (right) Box plot of TM-scores for structures predicted using AF for each TM-score threshold used in the design fitness function. Percentage of designs below the threshold is reported. The centre line of each box corresponds to the median, box edges to the upper and lower quartiles, and whiskers to the lowest and highest data points excluding outliers (within 1.5 times the interquartile range). (**C**) Box plot of predicted dissociation constants for a set of selected multi-state binder designs ($N = 20{,}000$ molecular dynamics snapshots per box). Designs are grouped by target protein and the structures of each target are displayed above. Boxes, centre line and whiskers as in (**B**).

 © The Author(s)
